# Reply to: Failure to apply standard limit-of-detection or limit-of-quantitation criteria to specialized pro-resolving mediator analysis incorrectly characterizes their presence in biological samples

**DOI:** 10.1038/s41467-023-41767-9

**Published:** 2023-11-10

**Authors:** Jesmond Dalli, Esteban A. Gomez

**Affiliations:** 1 William Harvey Research Institute, Barts and The London School of Medicine and Dentistry, Queen Mary University of London, Charterhouse Square, London, EC1M 6BQ UK; 2 Centre for Inflammation and Therapeutic Innovation, Queen Mary University of London, London, UK

**Keywords:** Immunology, Inflammation

**replying to** V. B. O’Donnell et al. *Nature Communications* 10.1038/s41467-023-41766-w (2023)

O’Donnell et al.^1^ assert that because they can achieve an area under the curve (AUC) of ≥2000 cps by integrating background noise, our criteria “lead to flawed … biomarker claims.” This assertion is based on the misapplication of the criteria that were described clearly, in our view, leading to erroneous results and therefore conclusions. In Gomez et al., the criterion described for peak identification and integration, and therefore calculation of the AUC, requires the presence of a distinct peak in the chromatogram as denoted by the following text in the methods: presence of a peak with a minimum area of 2000 counts. In the regions of chromatograms integrated by O’Donnell et al., there is no single discernible peak and therefore it would not meet the basic criterion for integration. To further clarify the application of the criteria employed in the analysis of data presented in Gomez et al.^2^, we provided an illustration that presents the decision pathway used for peak identification and integration (Supplementary Fig. 1). We believe that this aspect alone undermines O’Donnell et al.’s argument regarding the validity of our approach, since they do not demonstrate that blank samples yield a single discernible peak with an AUC ≥2000 cps. Nonetheless, and to further substantiate our argument, we also provide examples of chromatograms from our data analysis with a side-by-side comparison of AUC and signal-to-noise (s/n) ratios (see below and Supplementary Fig. 2). We present examples at the lower extreme of the identification spectrum, substantiating the argument that even the lower abundance peaks gave s/n ratios ≥5, with the signal obtained for most mediators identified being well above this threshold. In the Supplementary text we provide a discussion on the rationale behind the methodologies employed in Gomez et al.^2^.

O’Donnell et al. also argue that because we did not use s/n ratios as the cut-off parameter for determining the lower limits of quantitation (LLOQ) our analysis is flawed and that SPMs do not exist in biological systems. We respectfully disagree with this assertion and point of view. First, there is extensive documentation from many groups (including some of the co-authors of O’Donnell et al.^3–10^, also see the following recent reviews for a more comprehensive list of publications^10,11^) which identify and quantitate SPM in an array of biological systems. Second, while it is the case that the different entities mentioned by the authors have recommended the use of s/n ratios as an analytical criterion, this is not the only criterion they recommend.

Whilst we acknowledge that using s/n ratios is useful in determining the LLOQ/LLOD of LC-MS/MS assays, a review of the literature, including documents cited by O’Donnell et al., demonstrates that there are several approaches for the calculation of such parameters, and there are also different guidelines for cut-offs to be employed^12,13^. We note that independent of the approach used the methods need to be accurate and precise, aspects that are difficult to achieve with a pencil and ruler as employed by O’Donnell et al. in the reanalysis of our published dataset.

To demonstrate the robustness of the approach used in Gomez et al., we reanalyzed the underlying data from this publication using an orthogonal approach with the LLOQ set as a s/n ≥5. Due to (1) space limitations, (2) given that O’Donnell et al. claim that our approach does not support the utility of measuring SPMs as biomarkers and (3) since machine learning models are exquisitely sensitive to changes in the variables being used, in this response we show the results obtained from the reanalysis of data presented in Figure 1 of Gomez et al. Of note, both the accuracy and AUC values obtained using this orthogonal method gave essentially identical outcomes to those published in Gomez et al., supporting the robustness of the analysis performed in our publication (Fig. 1).

**Fig. 1:**
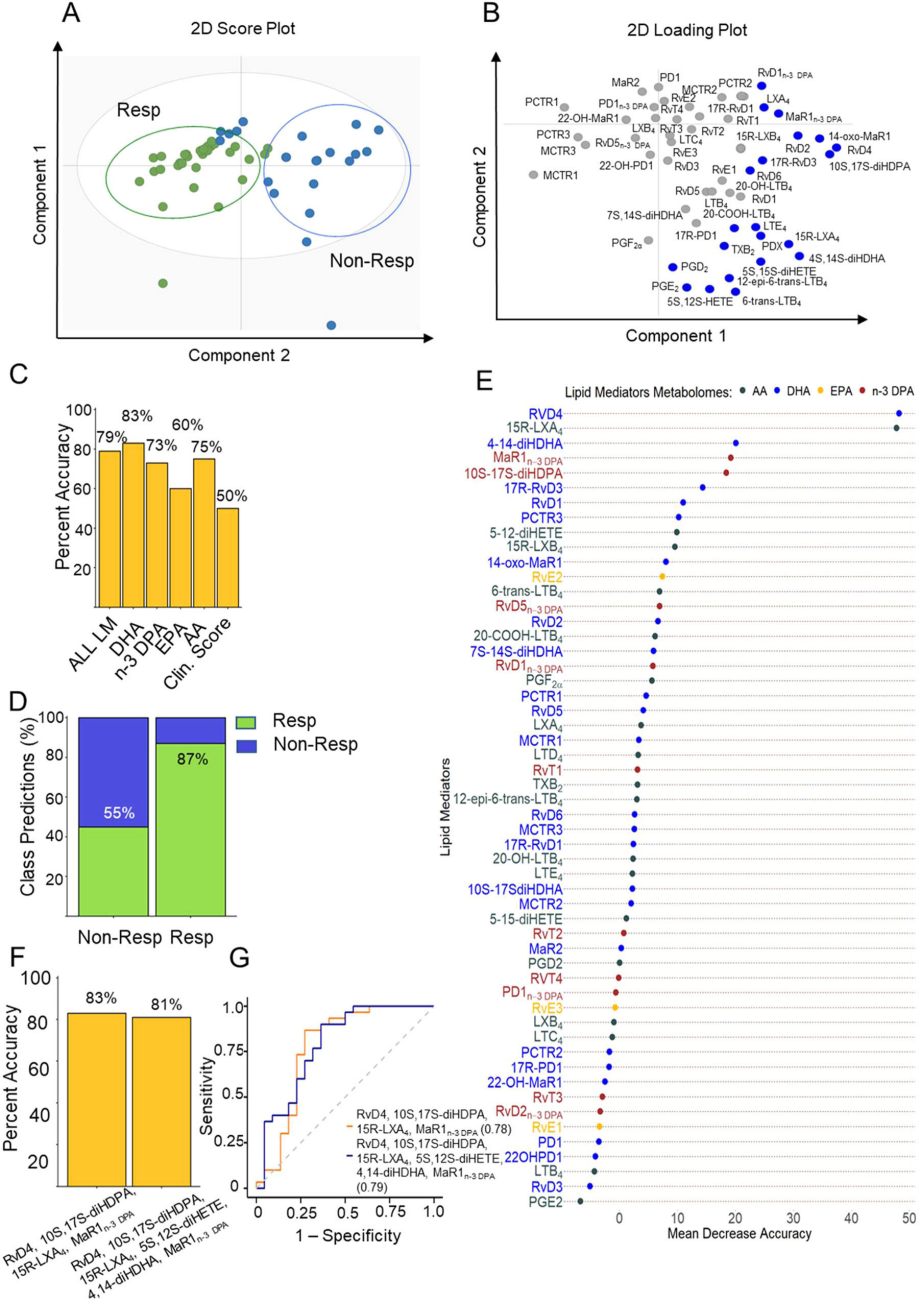
Reanalysis of baseline plasma lipid mediator profiles supports their potential utility as biomarkers. Plasma was collected from RA patients prior to the initiation of treatment with DMARDs and lipid mediator concentrations established using LC-MS/MS-based lipid mediator profiling (see Supplementary Methods for details). **A**, **B** OPLS-DA analysis of peripheral blood lipid mediator concentrations for DMARD responders (Resp) and DMARD non-Responders (Non-Resp). **A** Two-dimensional score plot with the gray circle representing the 95% confidence regions. **B** Two-dimensional loading plots. Lipid mediators with VIP score greater than 1 are highlighted in blue and upregulated in Non-Resp. Results are representative of *n* = 30 Resp and *n* = 22 Non-Resp. **C** Percentage accuracy score of prediction models based on the combination of all lipid mediators identified and quantified (AL LM) or individual fatty acid metabolomes as indicated. Clin. Score = clinical score (see methods from Gomez et al., for parameters included). **D** Classification predictions for each class (sensitivity and specificity) of the n-3 DPA model. Green indicates the samples that were predicted as Resp while blue indicates those patients predicted Non-Resp. Percentages indicate true positives (Resp class) and true negatives (Non-Resp class). **E** Relevance of lipid mediators in the prediction performance of the “ALL LM” model based on decreasing accuracy. **F** Percentage accuracy score of models using the indicated SPM. **G** Receiver operating characteristic (ROC) curves and AUC values for predictive models based on the indicated SPM. All the models were created using the random forest methodology (“randomForest” package from R).

The claim made by O’Donnell et al. that software developed by SCIEX yields inaccurate results would, in our view, require substantiation, especially since these authors appear to use SCIEX software for the calculation of s/n ratios in their publications^6,14,15^. We are also unclear of the scientific basis for the assertion that s/n should not be calculated after smoothing, especially because software from several vendors automatically performs this data-processing step. Furthermore, as noted elsewhere (SCIEX OS for Triple Quadrupole Systems Software User Guide [https://sciex.com/content/dam/SCIEX/pdf/customer-docs/user-guide/sciex-os-tnt-user-guide-en.pdf]) when applied appropriately smoothing increases the robustness of the s/n analysis by reducing the fluctuation in both the background signal and the signal for the peak of interest.

O’Donnell et al. also claim that they were able to obtain a spectrum that would match that of Maresin (MaR) 1 from a blank sample. We cannot know what contaminants there may have been in their instrumentation that may have contributed to their result. As can be observed from the data presented in Supplementary Fig. 3, the evaluation of blanks on our instrumentation did not yield MS/MS spectra that contained ions that could be linked with lipid mediator identification. Focusing on MaR1, when we extracted ions with an m/z of 359.4 corresponding to the MaR1 parent ion (and other dihydroxylated SPM derived from DHA) we did not observe any eluting at the retention time corresponding with that of MaR1 (and other dihydroxylated SPM). Furthermore, the evaluation of MS/MS spectra captured for molecules eluting before and after the retention time of MaR1 did not yield any of the ions reported by O’Donnell et al., suggesting that the spectra they reported arise from contaminants within their instrumentation. To further substantiate the utility of using MS/MS spectra for identification of SPM in our samples, we used the library match function in SCIEX OS. This analysis confirmed the presence of diagnostic MS/MS spectra in the plasma samples (Supplementary Fig. 4).

In summation, our view is that the critique made by O’Donnell and colleagues^1^ of our article is undermined by the misrepresentation of our criteria, potential contaminants in the system used, and by critical issues in the methodologies on which they are based. The reanalysis of the original data using orthogonal approaches and objective methodologies further substantiates both the presence of SPMs in human peripheral blood, in line with findings made by others^3,5,6,16–18^, and their utility as biomarkers. We welcome the opportunity to discuss results published in Gomez et al.^2^ and further demonstrate the strength of the identifications and conclusions.

## Methods

Data acquisition, multivariate analysis and machine learning models were performed as detailed in Gomez et al.^1^. For the calculation of s/n ratios the AutoPeak and Noise filtering, and Relative Noise functions in SCIEX OS v2.1 were employed. See Supplementary Methods.

### Reporting summary

Further information on research design is available in the Nature Portfolio Reporting Summary linked to this article.

### Supplementary information

Supplementary Information

Reporting Summary

## Data Availability

The data that support the figures and other findings within this paper are available from the corresponding authors upon request.
