# Supplementary material for: Reply to: Failure to apply standard limit-of-detection or limit-of-quantitation criteria to specialized pro-resolving mediator analysis incorrectly characterizes their presence in biological samples
Source: Nat Commun. 2023 Nov 10;14:7293. doi: 10.1038/s41467-023-41767-9 (PMC10638266; doi:10.1038/s41467-023-41767-9)

Supplementary Information

Reply to: 'Failure to apply standard limit-of-detection or limit-of-quantitation criteria to specialized pro-resolving mediator analysis incorrectly characterizes their presence in biological samples.'

Jesmond Dalli[1,2] and Esteban A. Gomez[1]

[1] William Harvey Research Institute, Barts and The London School of Medicine and Dentistry, Queen Mary University of London, Charterhouse Square, London, EC1M 6BQ UK.

[2] Centre for Inflammation and Therapeutic Innovation, Queen Mary University of London, London, UK

Supplementary Text

A complicating factor that all workers in this field of lipid mediators and natural small molecules are acutely aware of is the presence of several isobaric isomers which elute in the proximity of the mediators of interest. These isomers interfere with the calculation of s/n ratios when using classic approaches, since their presence precludes the identification of an appropriate region within the chromatogram that represents a true baseline, and which is therefore sufficiently wide and close to the peak of interest to accurately calculate the value for the 'noise'.

To overcome this limitation, we developed a method that retains the required robustness and is not hampered by the presence of biological isomers within the chromatogram. In support of the robustness of our method we provided tables in our publication where we present the coefficient of variations on the two instruments we employed for the analysis (Supplementary Tables 11 and 12 from Gomez *et al* [1]). O'Donnell *et al*, may have overlooked this key dataset.

Since the publication of our article software-based approaches to overcome this issue have become available. Amongst these is the solution offered in Sciex OS and referred to as the 'relative noise' algorithm. This algorithm calculates the baseline under the peak of interest by calculating the noise in the chromatographic region. This approach overcomes many of the issues classically associated with s/n calculations, including the subjective nature of the noise region selection and the presence of other peaks within the region of interest that make it challenging to identify a sufficiently wide 'noise' region to obtain a correct estimate. The following documentation details this concept and the underlying algorithm (SCIEX OS for

Triple Quadrupole Systems Software User Guide). Given these features, we selected to employ this methodology in our re-analysis.

O'Donnell *et al*. also assert that the MS/MS criteria using in Gomez *et al*. employed in the identification of SPMs were flawed and that the ions employed in the identification were derived from background noise. They provide MS/MS spectra that were generated in their laboratory using different instrumentation, and likely different instrument parameters, and then proceed to use these as reference spectra for their argument. They further used MS/MS spectra from a reference database which was compiled using completely different mass spectrometers, and different acquisition parameters including different cut-off values for the MS/MS spectrum (which go below an m/z of 100), to further bolster their argument. We are surprised that the authors deem this to be an appropriate approach given that they themselves acknowledge in their article that different mass spectrometers are likely to yield distinct MS/MS spectra. We therefore find this argument to be flawed.

Supplementary Methods

Data acquisition, multivariate analysis and machine learning models were performed as detailed in Gomez *et al*. For the calculation of signal-to-noise ratios the AutoPeak and Noise filtering, and Relative Noise functions in Sciex OS v2.1 were employed.

To amplify and support the utility of MS/MS spectra in the identification of SPM, we reanalyzed the underlying the data presented in Gomez *et al*. employing the library match function in Sciex OS, whereby the software matches the ions present in the samples with those in the reference spectrum and provides a score. Here we used a score of ≥70% and matching retention time as a confirmation of a positive match. As can be appreciated in the examples provided in Supplemental Figure 4, this reanalysis confirmed the initial observations published in Gomez *et al*. that many of the SPMs are present in plasma from RA patients.

Supplementary References

1. Gomez, E.A., Colas, R.A., Souza, P.R. *et al.* Blood pro-resolving mediators are linked with synovial pathology and are predictive of DMARD responsiveness in rheumatoid arthritis. *Nat Commun* **11**, 5420 (2020). https://doi.org/10.1038/s41467-020-19176-z

Supplementary Figures Legends

Supplementary Figure 1: Step-by-step illustration of the criteria for peak integration.

Supplementary Figure 2: Representative examples of peaks corresponding to distinct mediators identified in Gomez *et al*. In these examples we also denote the AUC values and the corresponding sign-to-noise ratios. Furthermore, one can also appreciate the presence of several biological isomers that elute in close proximity to many of the peaks of interest.

Supplementary Figure 3: Blank samples do not yield MS/MS spectra of diagnostic value. (A) Screenshot of MaR1 standard denoting the retention time of this mediator (B-D). A blank sample was injected and the (B) extracted total ion chromatogram for m/z 359.4 corresponding to the MaR1 parent ion was obtained. (C) MS/MS spectrum obtained from the signal reported at a retention time of 13.93 in the ion chromatogram reported in (B) demonstrating the absence of diagnostic ions.

Supplementary Figure 4: Matching of MS/MS spectra from obtained from RA patient plasma samples using the library function in Sciex OS confirms the presence of these autacoids. MS/MS spectra obtained from plasma samples described in Gomez *et al*, were matched using the library function in Sciex OS and a threshold value >70% score.

Supplementary Figure 1

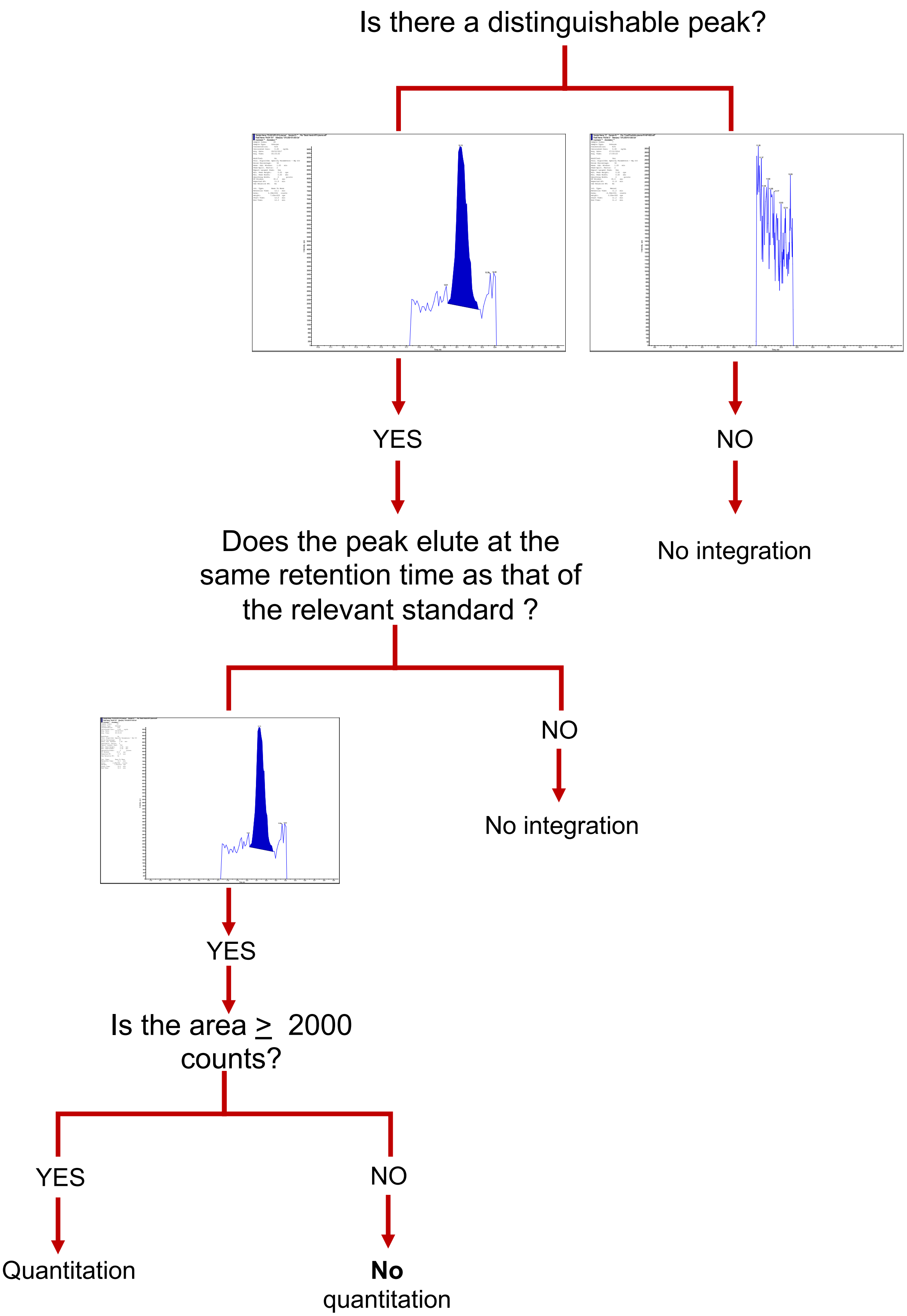

Supplementary Figure 2

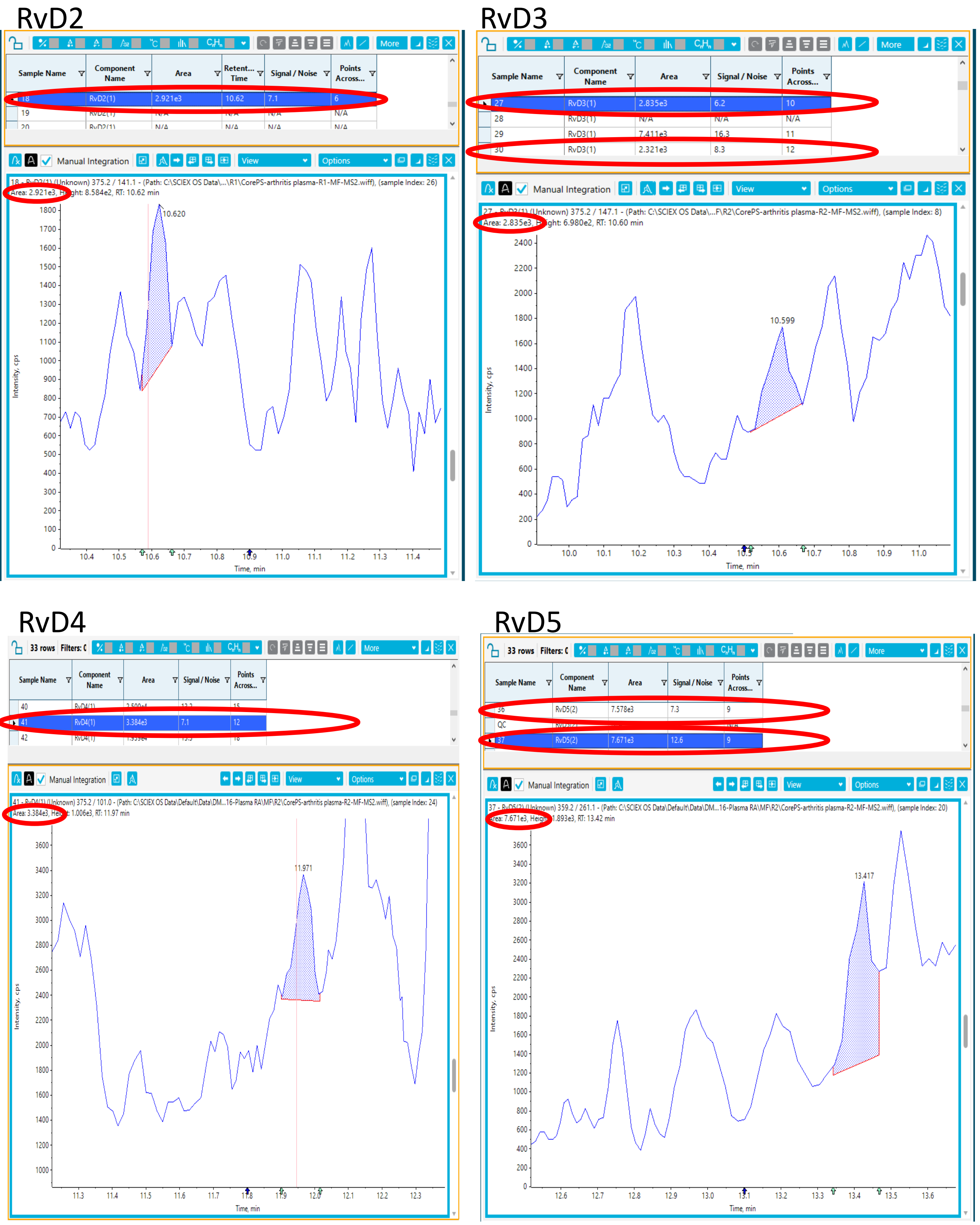

Supplementary Figure 2

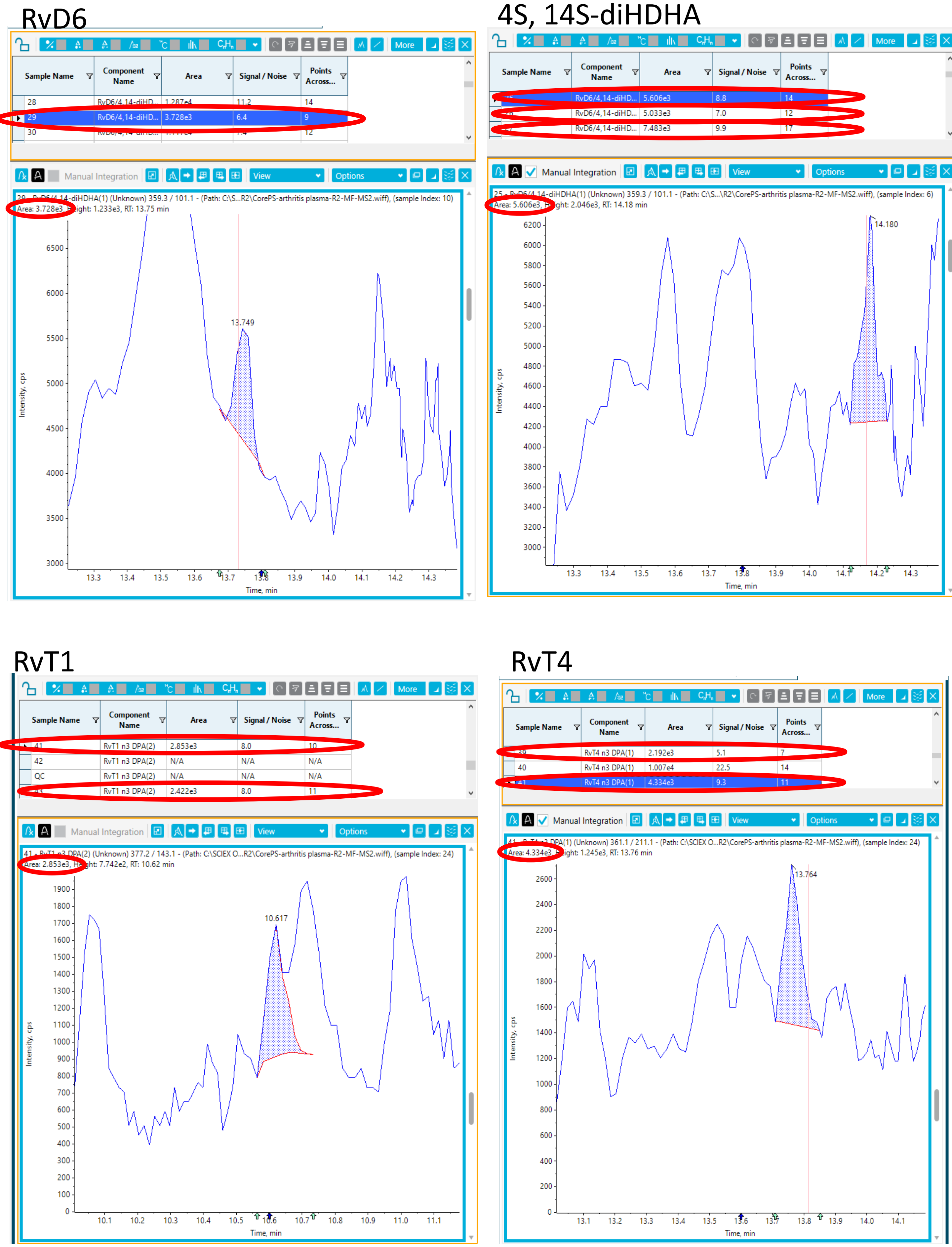

Supplementary Figure 2

PD1n-3 DPA

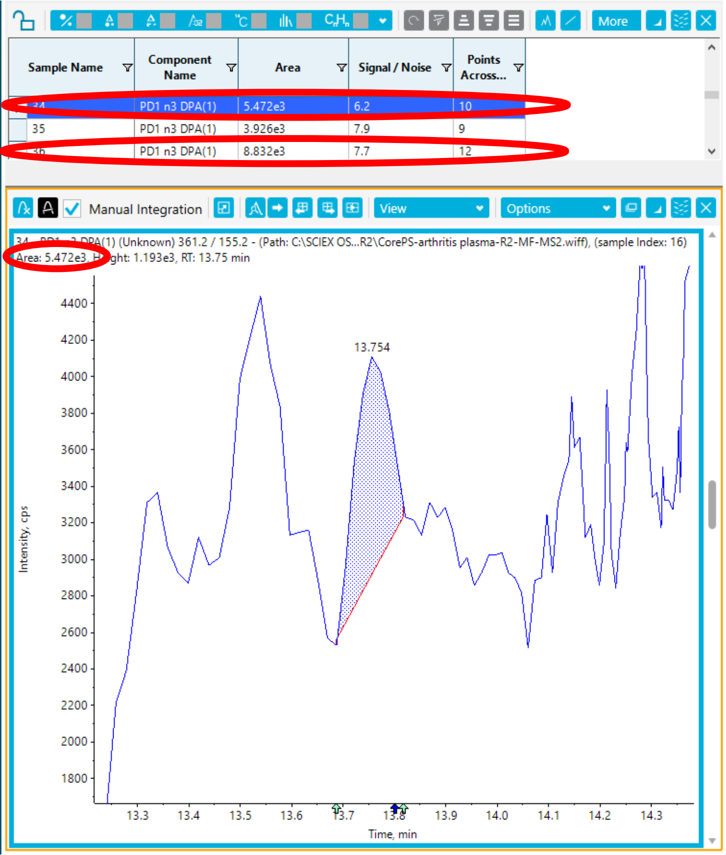

RvD1n-3 DPA

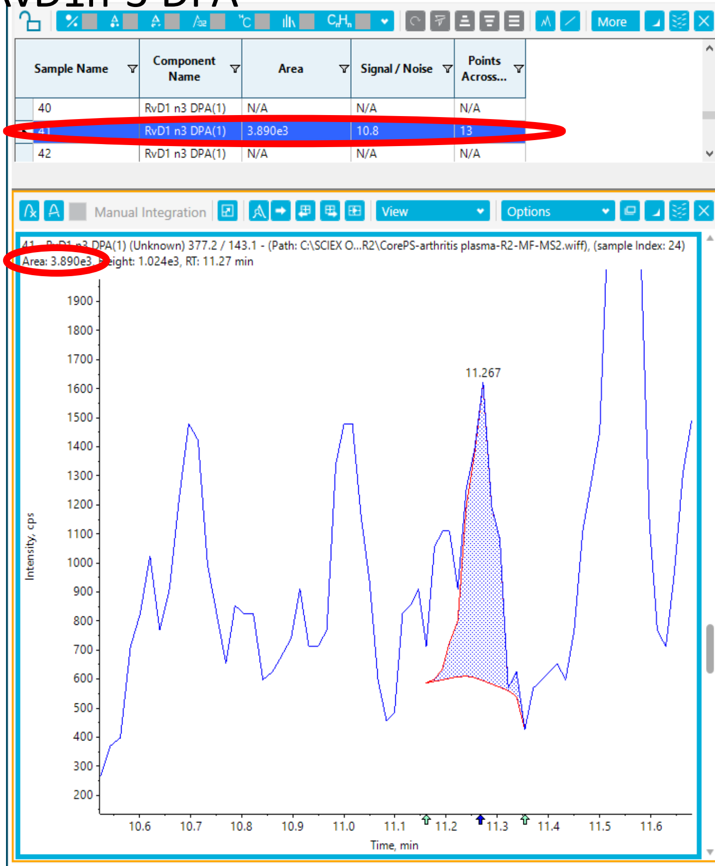

RvD5$_{n-3\ DPA}$

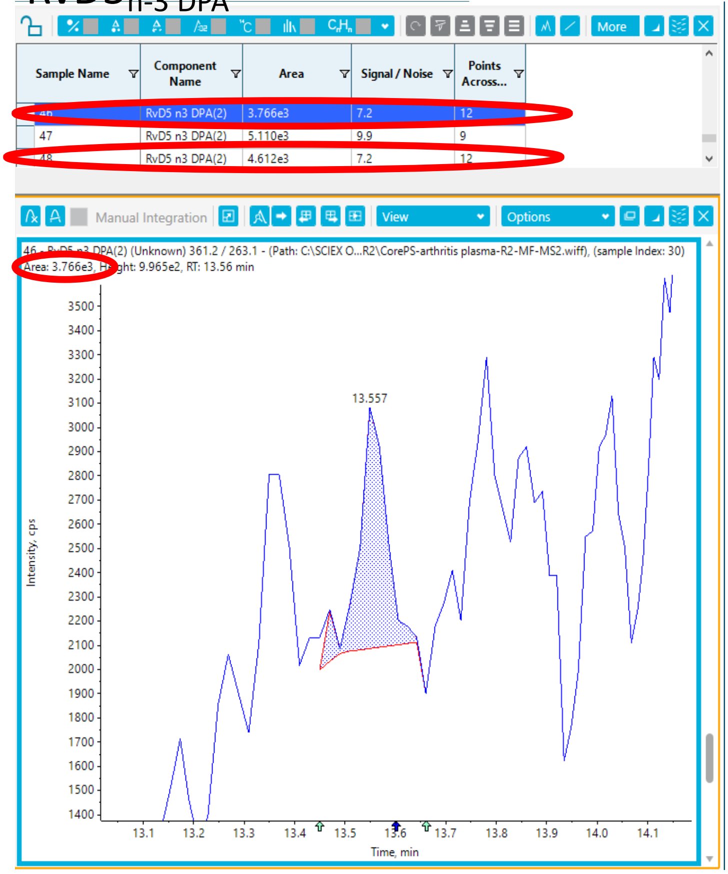

MaR1$_{n-3\ DPA}$

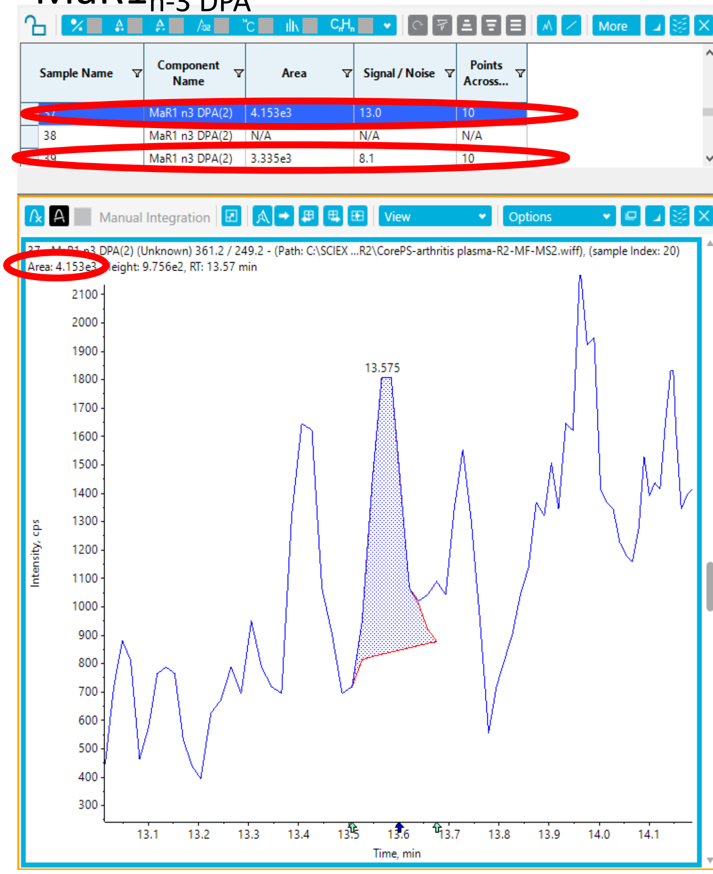

Supplementary Figure 2

RvE1

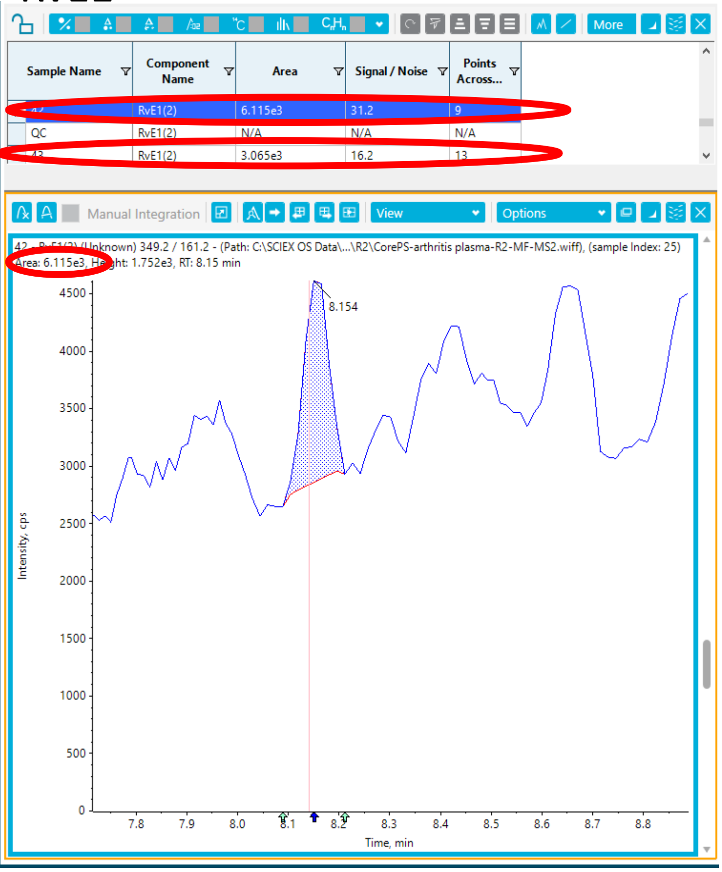

LXB_4

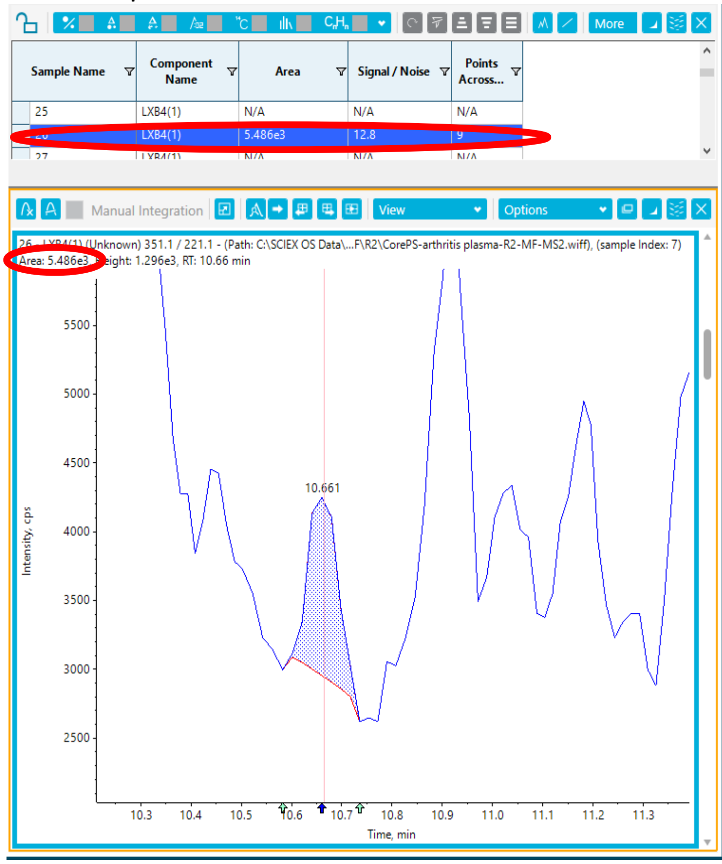

10S, 17S-diHDPA

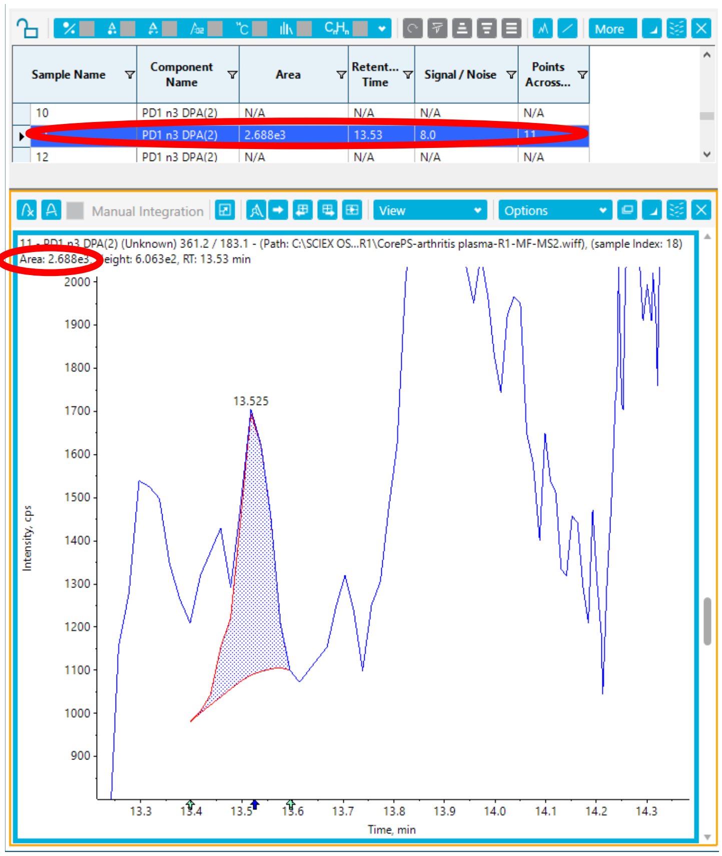

14-oxo-MaR1

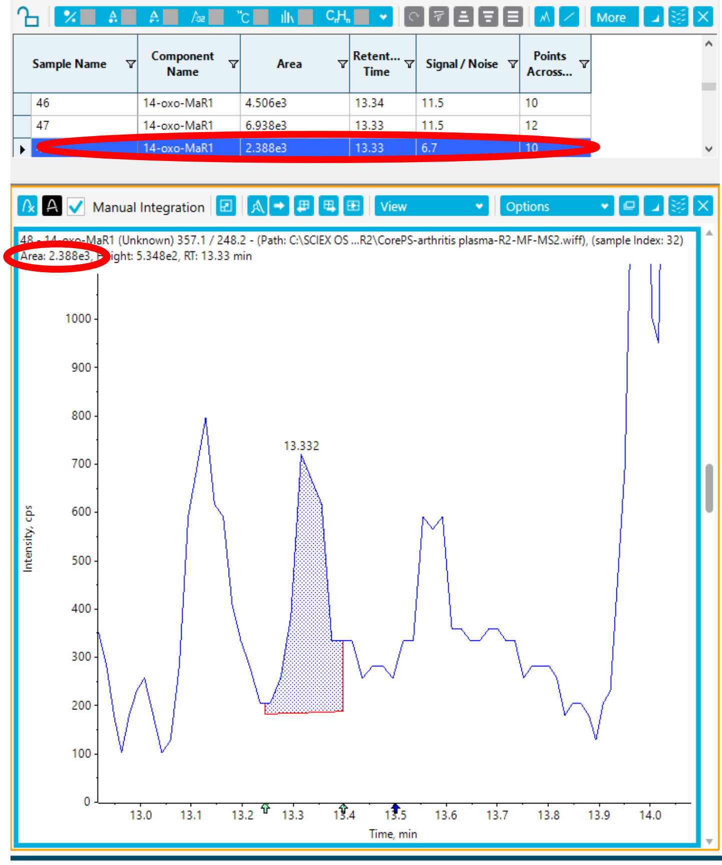

Supplementary Figure 3

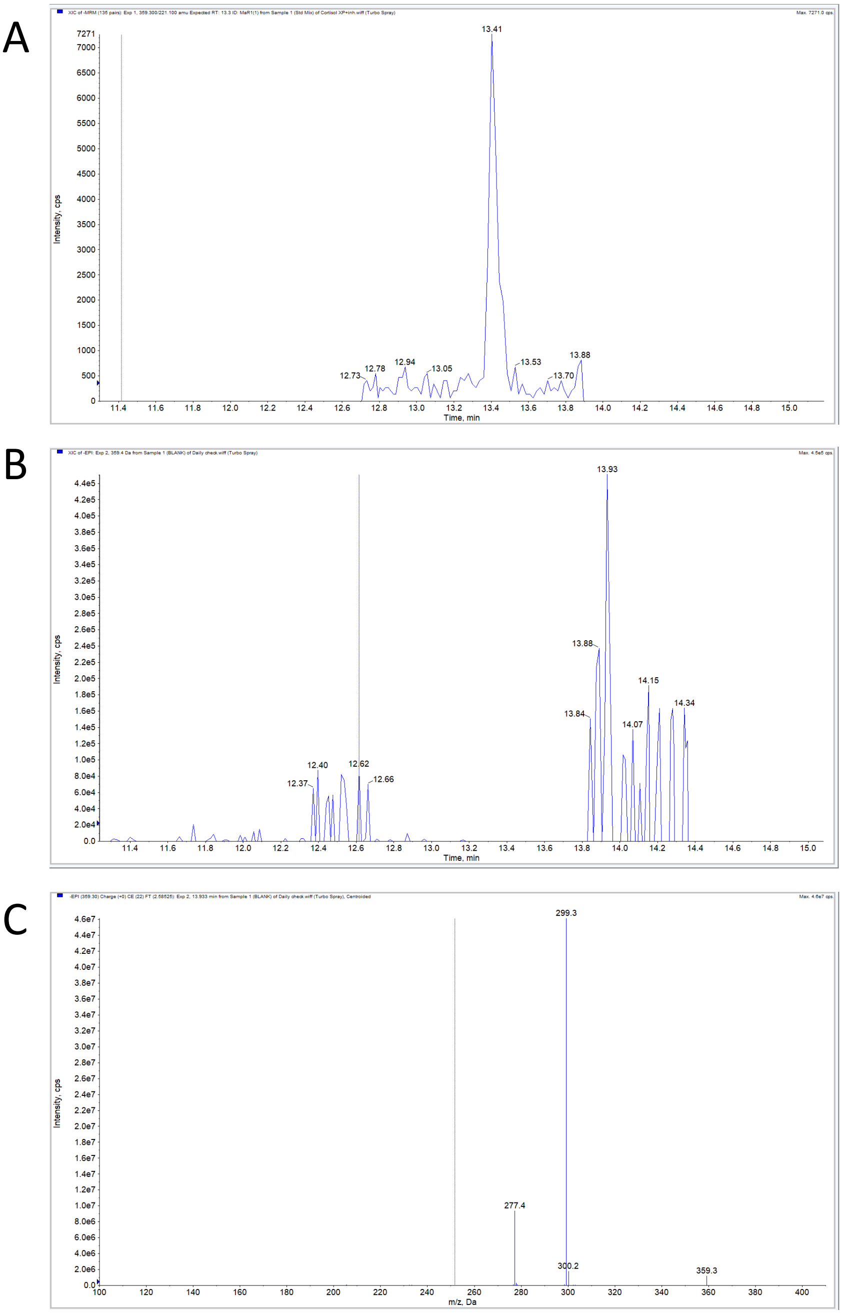

Supplementary Figure 4

RvD1

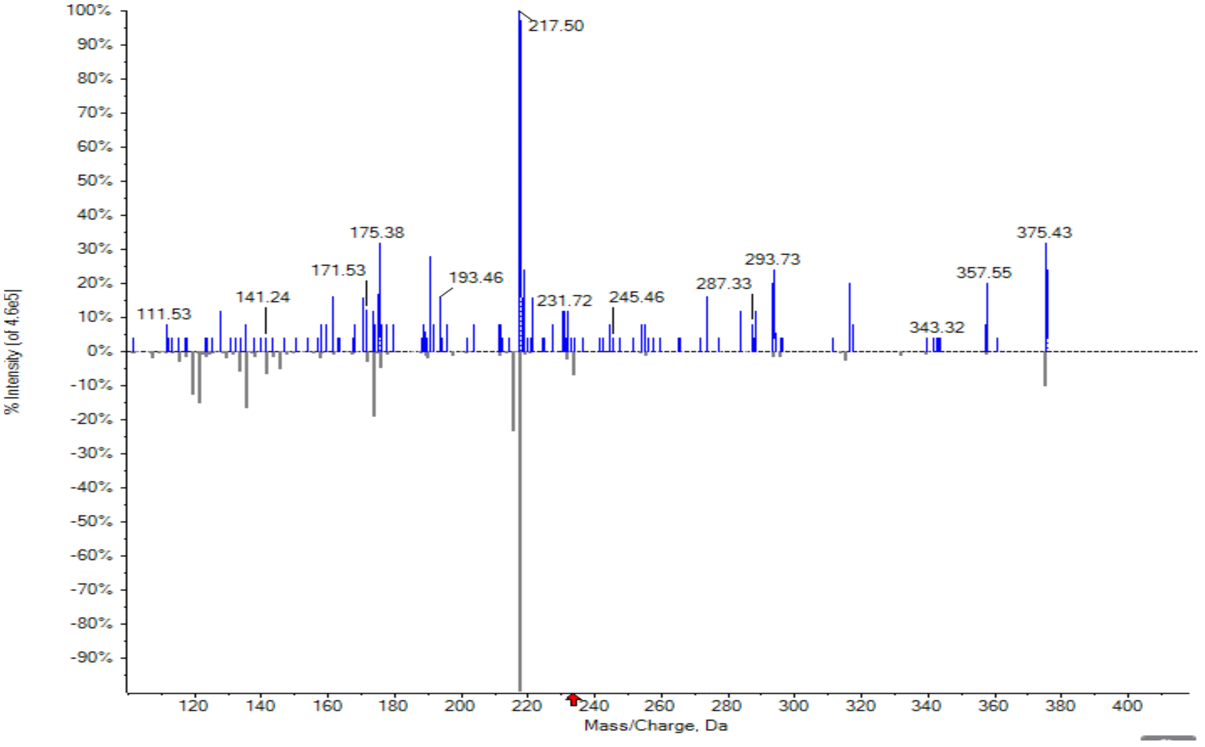

RvD4

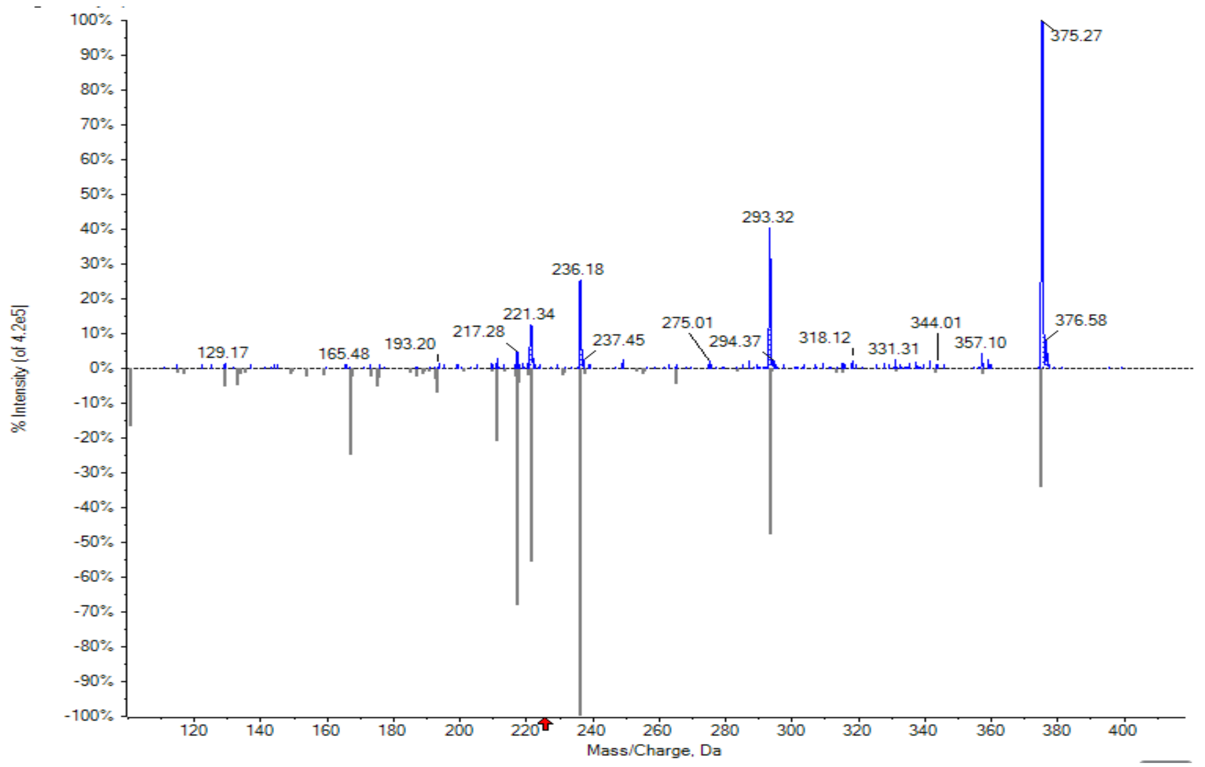

Supplementary Figure 4

RvD2

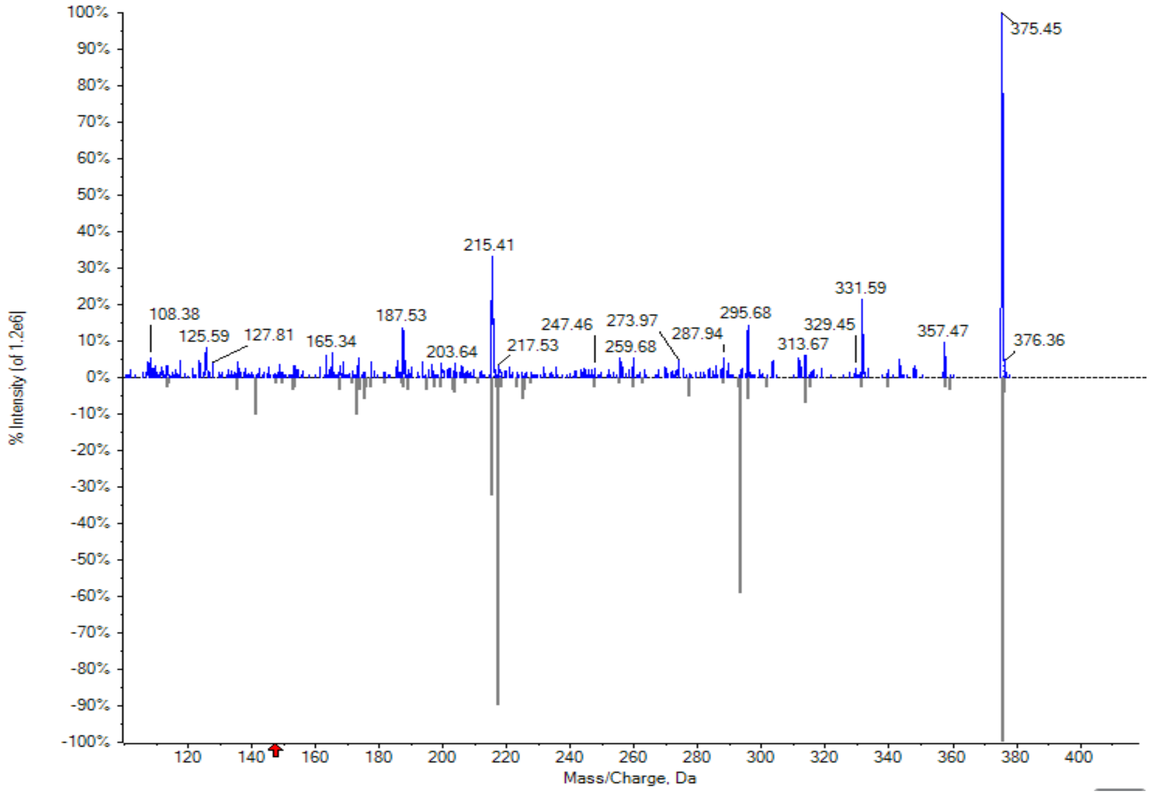

RvE3

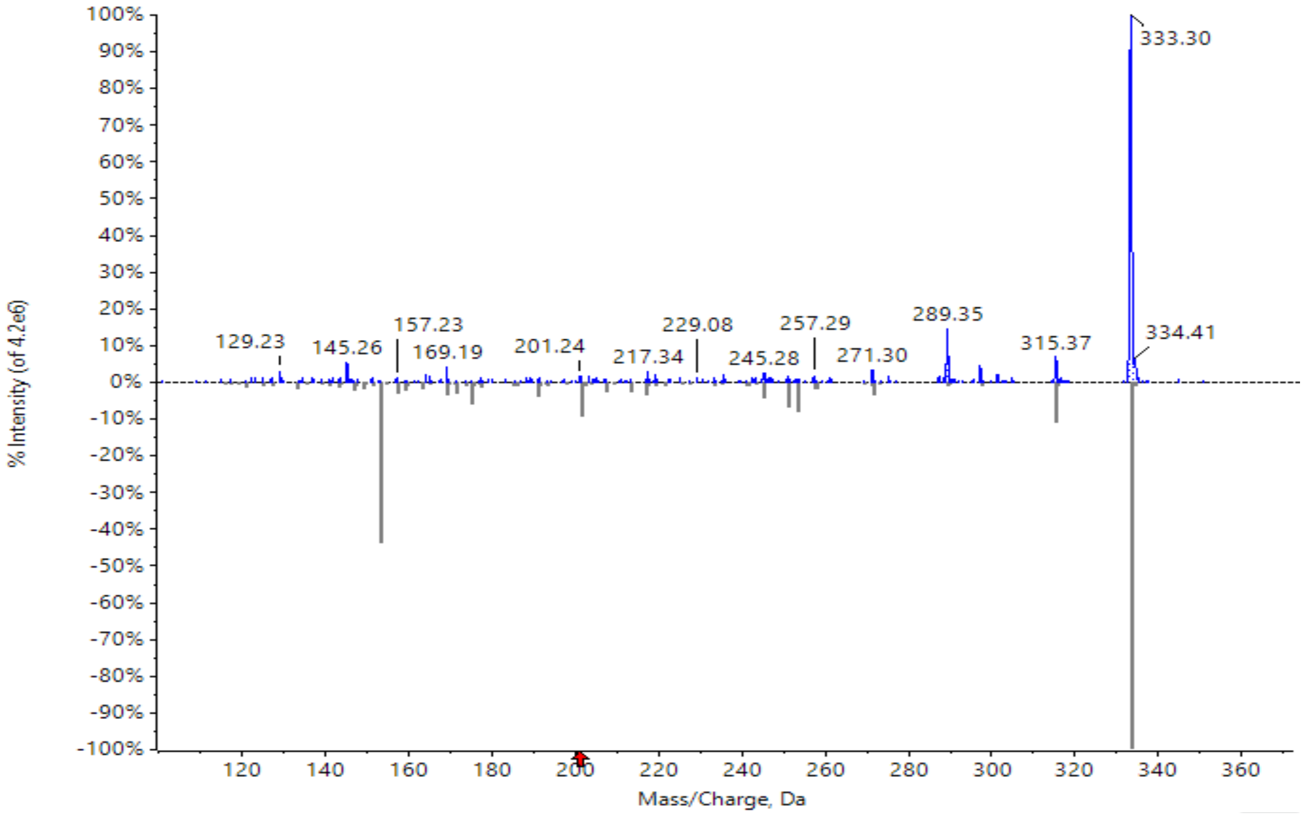

Supplementary Figure 4

LXA$_4$

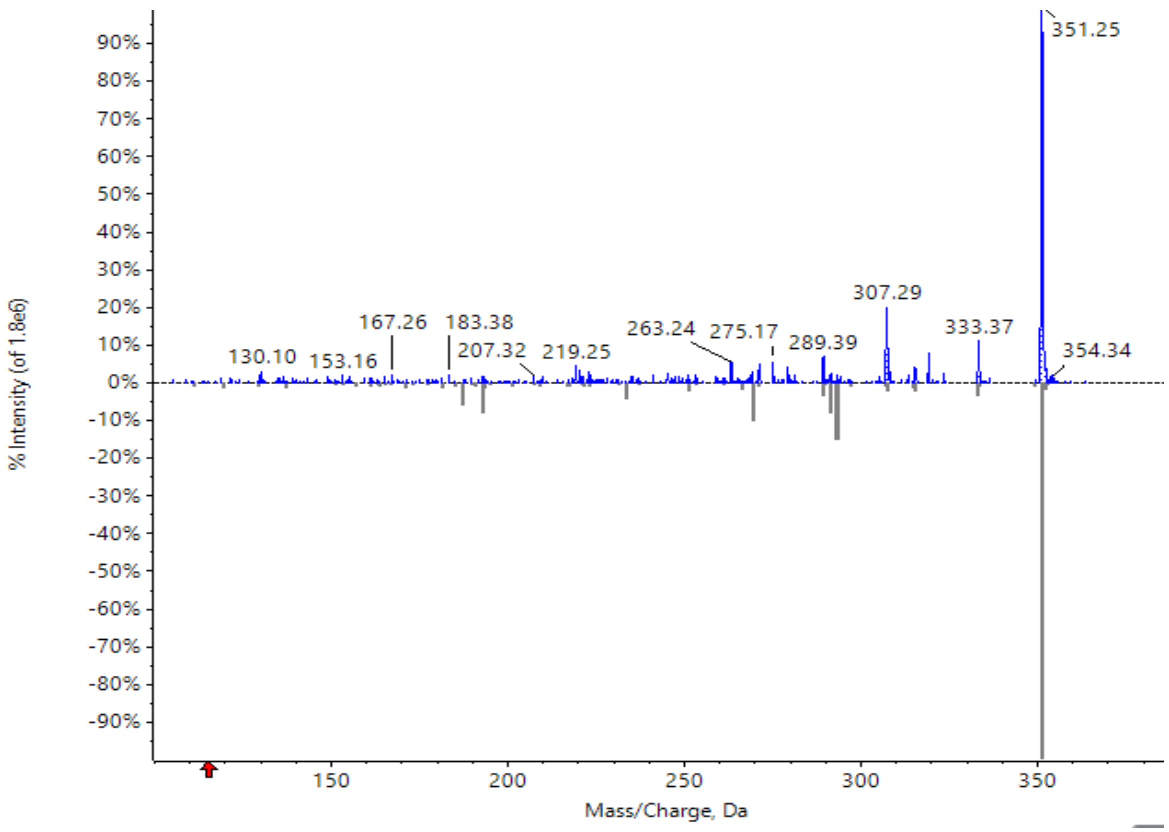

MaR1

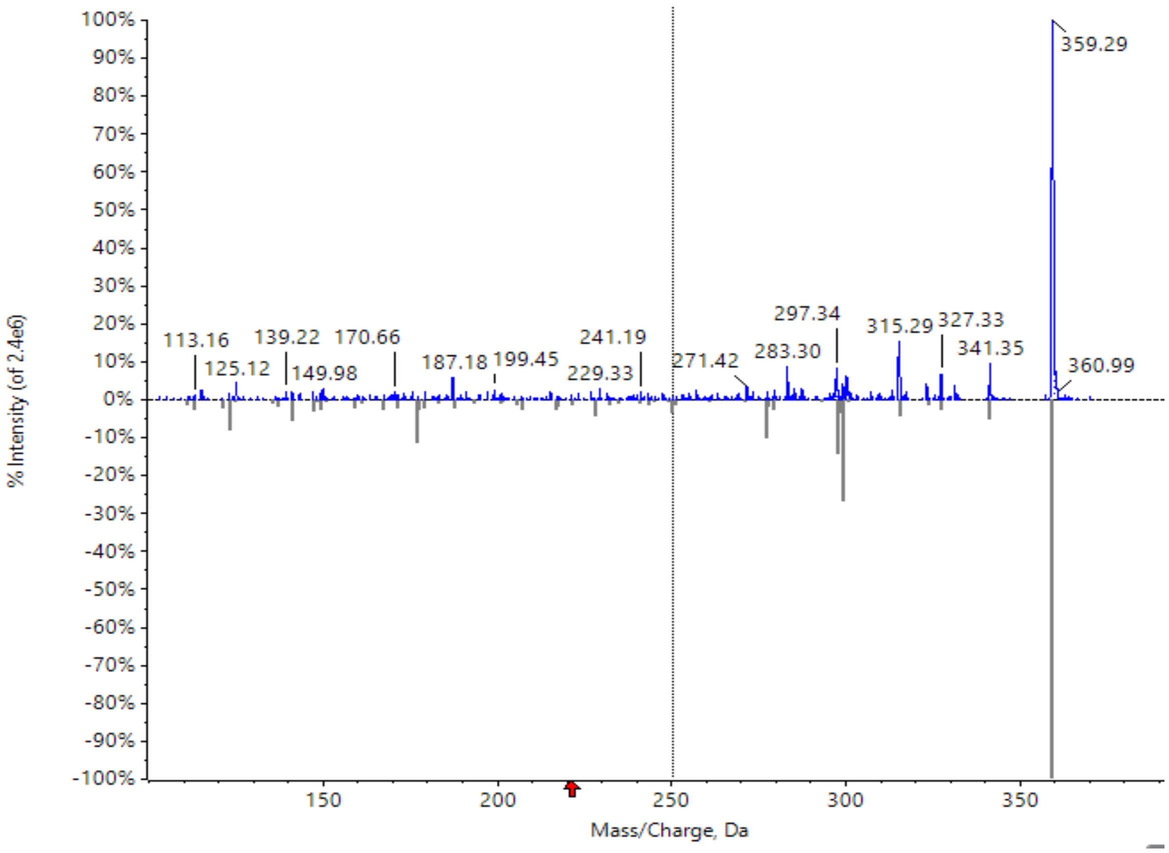

Supplement: Supplementary file 1 — Supplementary Information [file 41467_2023_41767_MOESM1_ESM.pdf]
