# Supplementary material for: Reply to: Failure to apply standard limit-of-detection or limit-of-quantitation criteria to specialized pro-resolving mediator analysis incorrectly characterizes their presence in biological samples
Source: Nat Commun. 2023 Nov 10;14:7293. doi: 10.1038/s41467-023-41767-9 (PMC10638266; doi:10.1038/s41467-023-41767-9)
Supplement: Supplementary file 2 — Reporting Summary [file 41467_2023_41767_MOESM2_ESM.pdf]

nature portfolio

Corresponding author(s): Jesmond Dalli
Last updated by author(s): Aug 15, 2023

Reporting Summary

Nature Portfolio wishes to improve the reproducibility of the work that we publish. This form provides structure for consistency and transparency in reporting. For further information on Nature Portfolio policies, see our Editorial Policies and the Editorial Policy Checklist.

Statistics

For all statistical analyses, confirm that the following items are present in the figure legend, table legend, main text, or Methods section.

n/a	Confirmed	
☐	☒	The exact sample size (*n*) for each experimental group/condition, given as a discrete number and unit of measurement
☐	☒	A statement on whether measurements were taken from distinct samples or whether the same sample was measured repeatedly
☒	☐	The statistical test(s) used AND whether they are one- or two-sided<br>*Only common tests should be described solely by name; describe more complex techniques in the Methods section.*
☒	☐	A description of all covariates tested
☒	☐	A description of any assumptions or corrections, such as tests of normality and adjustment for multiple comparisons
☒	☐	A full description of the statistical parameters including central tendency (e.g. means) or other basic estimates (e.g. regression coefficient) AND variation (e.g. standard deviation) or associated estimates of uncertainty (e.g. confidence intervals)
☒	☐	For null hypothesis testing, the test statistic (e.g. *F*, *t*, *r*) with confidence intervals, effect sizes, degrees of freedom and *P* value noted<br>*Give P values as exact values whenever suitable.*
☒	☐	For Bayesian analysis, information on the choice of priors and Markov chain Monte Carlo settings
☒	☐	For hierarchical and complex designs, identification of the appropriate level for tests and full reporting of outcomes
☒	☐	Estimates of effect sizes (e.g. Cohen's *d*, Pearson's *r*), indicating how they were calculated

Our web collection on statistics for biologists contains articles on many of the points above.

Software and code

Policy information about availability of computer code

Data collection	Analyst v1.63 (Lipid mediator profiling),
Data analysis	Sicex OS Q v2.1 and R Software. The relevant code can be found Github repository https://github.com/eagomezc/2019_Machine_Learning_DMARD_in_RA_patients

For manuscripts utilizing custom algorithms or software that are central to the research but not yet described in published literature, software must be made available to editors and reviewers. We strongly encourage code deposition in a community repository (e.g. GitHub). See the Nature Portfolio guidelines for submitting code & software for further information.

Data

Policy information about availability of data

All manuscripts must include a data availability statement. This statement should provide the following information, where applicable:

- Accession codes, unique identifiers, or web links for publicly available datasets
- A description of any restrictions on data availability
- For clinical datasets or third party data, please ensure that the statement adheres to our policy

Access requests to the data can should be addressed to the lead author.

Research involving human participants, their data, or biological material

Policy information about studies with human participants or human data. See also policy information about sex, gender (identity/presentation), and sexual orientation and race, ethnicity and racism.

Reporting on sex and gender	Samples were collected from both males and females.
Reporting on race, ethnicity, or other socially relevant groupings	Data on race, ethnicity and other socially relevant groupings was not collected
Population characteristics	RA patients fulfilling 2010 American College of Rheumatology/European League Against Rheumatism (EULAR) Classification Criteria, with clinically defined synovitis and symptom duration less than 12 months, were enrolled as part of the 'Pathobiology of Early Arthritis Cohort' (PEAC, http://www.peac-mrc.mds.qmul.ac.uk) at three UK Academic Centres: Queen Mary University of London/Barts Health NHS trust, University of Glasgow and University of Birmingham. All patients were naïve to steroid and DMARD therapy.
Recruitment	Patients underwent a ultrasound (US)-guided synovial biopsy (figure 1A) procedure we pioneered6 of a clinically active joint selected according to a previously defined algorithm to ensure maximal synovial tissue retrieval6; a minimum of 12 synovial biopsies were stored for subsequent analysis at the William Harvey research Institute (six for histological analysis and six for RNA extraction), and patients then commenced on standard DMARD therapy and/or low-dose corticosteroid. A treat-to-target approach to therapy escalation was followed aiming for low disease activity score-28 (DAS28) <3.2. Patients failing DMARD therapy were commenced on biological therapy according to the UK National Institute for Clinical Excellence prescribing algorithm for RA patients if they continued to have a DAS28 >5.1 at 6 months.<br>Ultrasonographic images were collected at the time of biopsy for both the individual biopsied joint and the global joint score: first to fifth metacarpophalangeal (MCP) joints and midline, radial and ulnar views of both wrist joints. Images subsequently underwent semiquantitative assessment by a single blinded (to clinical/histological data) assessor for both synovial thickening (ST) and power Doppler (PD) activity according to standard EULAR-OMERACT (Outcome Measures in Rheumatology) US synovitis scores (grade 0–3).7 The mean global ST and PD scores including the maximal score for the wrist joint were then determined.<br><br>Plain radiographs of the hands and feet performed at baseline and 12-month follow-up were scored in time sequential order according to the van der Heijde modified Sharp score (SHSS) by a single reader blinded to all clinical/histological data. The study received local ethical approval (REC-05/Q0703/198) and all participants gave written informed consent.
Ethics oversight	Ethics approval was obtained from the NHS REC

Note that full information on the approval of the study protocol must also be provided in the manuscript.

Field-specific reporting

Please select the one below that is the best fit for your research. If you are not sure, read the appropriate sections before making your selection.

- [x] Life sciences
- [ ] Behavioural & social sciences
- [ ] Ecological, evolutionary & environmental sciences

For a reference copy of the document with all sections, see nature.com/documents/nr-reporting-summary-flat.pdf

Life sciences study design

All studies must disclose on these points even when the disclosure is negative.

Sample size	Sample sizes for human peripheral plasma lipid mediator profiling were determined based on results obtained from published studies.
Data exclusions	N/A
Replication	For machine learning models, these were validated using an independent samples and the MCC scores for the predictability of the model were use to determine whether the results were replicable. In vitro and in vivo experiments were conducted on at least 2 separate occasions.
Randomization	N/A
Blinding	Investigators were blinded to group assignment for data acquisition and initial data analysis.

Reporting for specific materials, systems and methods

We require information from authors about some types of materials, experimental systems and methods used in many studies. Here, indicate whether each material, system or method listed is relevant to your study. If you are not sure if a list item applies to your research, read the appropriate section before selecting a response.

Materials & experimental systems

n/a	Involved in the study
☒	☐ Antibodies
☒	☐ Eukaryotic cell lines
☒	☐ Palaeontology and archaeology
☒	☐ Animals and other organisms
☒	☐ Clinical data
☒	☐ Dual use research of concern
☒	☐ Plants

Methods

n/a	Involved in the study
☒	☐ ChIP-seq
☒	☐ Flow cytometry
☒	☐ MRI-based neuroimaging
